# Risk Factors for Fragility Fractures in Chronic Lymphocytic Leukemia

**DOI:** 10.7759/cureus.54774

**Published:** 2024-02-23

**Authors:** Lloyd Petty, Deborah Stephens, Anu Sharma

**Affiliations:** 1 Endocrinology, Diabetes, and Metabolism, University of Utah School of Medicine, Salt Lake City, USA; 2 Hematology and Hematologic Malignancies, Huntsman Cancer Institute, University of Utah School of Medicine, Salt Lake City, USA; 3 Endocrinology, Diabetes, and Metabolism, University of Florida, Gainesville, USA

**Keywords:** secondary osteoporosis, generalized osteopenia, chronic lymphocytic leukemia (cll), dual energy x-ray absorptiometry, fragility fracture, bone mineral density, s: osteoporosis

## Abstract

Abnormal bone health and fragility fractures (FF) are more common in patients with chronic lymphocytic leukemia (CLL). We hypothesize that there may be risk factors in CLL patients with osteoporosis that increase the risk of FFs. We conducted a cohort study encompassing all patients diagnosed with CLL from January 1, 2000, to July 31, 2020, utilizing International Classification of Diseases (ICD) codes related to abnormal bone health (osteopenia, osteoporosis, and/or presence of FF) within a single tertiary care institution. Of the 89 patients included, 55 (62%) were female with a mean age of 68 ± 11 years at cohort entry. Fifty-nine (66%) had at least one FF present (pFF) and 30 (34%) did not have an FF (nFF). There were no differences in IGHV (Immunoglobulin heavy chain variable region gene) mutation status, chromosomal abnormalities, or the presence of a complex karyotype. The spine accounted for 81% of identified FF. *T*-score <-2.5 was more common in those without FF (pFF 38% vs. nFF 71%, *P *= 0.02). DXA evaluation was not conducted for 36 (40%) individuals within the cohort. Risk factors for fragility fractures included male sex (relative risk [RR] 8.1, 95% confidence interval [CI] 2.1-31.7), diabetes mellitus (RR 1.4, 95% CI 1.04-1.8), smoking (RR 1.3, 95% CI 1.02-1.8), Rai stage >0 (RR 1.4, 95% CI 1.04-1.9), and *T*-score >-2.5 (RR 1.8, 95% CI 1.1-3.1). There is a high frequency of vertebral FFs in people with CLL despite *T*-scores not being in the osteoporotic range. Increased awareness to screen and treat vertebral FFs in people with CLL is needed.

## Introduction

Chronic lymphocytic leukemia (CLL) and osteoporosis are diseases prevalent among older adults, characterized by high occurrence rates [1,2]. The pathophysiology of osteoporosis is characterized by uncoupling of osteoblasts and osteoclasts resulting in disruption in the bone architecture [3]. In CLL, there is infiltration of malignant cells into the bone marrow, increasing bone resorption and demineralization [4]. CLL, therefore, can cause bone loss and can exacerbate previously existing osteoporosis [4]. Despite this, there are limited clinical data describing the association of CLL with osteoporosis and fracture risk. 

In a cross-sectional study, 49% of patients diagnosed with CLL had bone loss on dual-X-ray absorptiometry (DXA) [5]. CLL was also associated with an excess risk of axial fractures, with a hazard ratio (HR) of 1.56 (95% CI 1.33-1.83) for pelvic fractures and HR of 1.29 (95% CI 1.16-1.44) for vertebral fractures [6]. In another study, however, there was no difference in the rate of vertebral compression fractures between people with CLL and controls [7]. Risk factors for vertebral fractures were abnormal T-scores, absolute lymphocyte count (ALC), and anemia [7].

Given that both CLL and osteoporosis affect similar age groups and can synergistically worsen bone health, we sought to (1) determine the risk factors for fragility fractures (FFs) in patients with CLL and abnormal bone health and (2) characterize the assessment of bone health in people with CLL and abnormal bone health.

## Materials and methods

Following approval from the Institutional Review Board (IRB# 00136464), we conducted an electronic search of the Huntsman Cancer Institute (HCI) CLL database. This search included all patients diagnosed with CLL and abnormal bone health, defined as diagnoses of osteopenia, osteoporosis, and/or a history of FFs, from January 1, 2000, to July 31, 2020, who received care at the HCI. Patients included in the study were identified from the CLL database with ICD-9 and ICD-10 codes associated with osteopenia, osteoporosis, and/or the presence of FF (M80.0, M81*, M85.8, M48.4, M84.35, M84.559, S72.00, and S52.5). Individuals were excluded if data on either CLL or bone health were missing. Demographic, clinical, laboratory, and radiologic data were extracted from the electronic medical record. Data corresponded to the time closest to the initial diagnosis of CLL or the first assessment of CLL at HCI.

The diagnosis of CLL was defined as ICD-9 and ICD-10 diagnosis codes for CLL (204.1 and C91*), a chart review of the treating oncologist’s clinic notes confirming the diagnosis of CLL, and laboratory data that were consistent with a diagnosis of CLL. The absolute lymphocyte count (ALC) was calculated using the following formula: ALC = (%lymphocytes + variant lymphocytes) x total white blood cell count (WBC). Rai staging was determined through chart review or independent calculation if not available in the clinical notes [8]. Lactate dehydrogenase (LDH) was reported as elevated once it was above the upper limit of normal for the assay performed. Bone mineral density (BMD) was measured using DXA. The machines and coefficients of variation varied among participants. If available, the images were reviewed by the authors. Definitions of normal BMD, osteopenia, and osteoporosis were established as follows: T-score > -1 was considered normal, between -1 and -2.5 indicated osteopenia, and <-2.5 signified osteoporosis, aligning with the World Health Organization (WHO) criteria [9]. An FF was defined as a fracture of the spine, wrist, or hip that occurred spontaneously or with minimal trauma. Participants were divided into two groups based on whether an FF was present (pFF) or absent (nFF) as documented by clinical notes and radiology reports (X-ray spine, CT spine, and MRI spine).

Statistical analysis

Statistical analysis was performed using JMP software v16. GraphPad version 7 was used for the graphical representation of data. Categorical variables were presented as percentages and analyzed using Pearson's Chi-squared or Fisher’s exact test when applicable. Continuous variables were expressed as mean with standard deviation (SD) when normally distributed and subjected to the two-sample t-test. Continuous variables with a non-Gaussian distribution were reported as median with interquartile range (IQR 25th-75th) and analyzed with nonparametric testing (Wilcoxon rank-sum test). Relative risk ratios (RRs) and their 95% confidence intervals (CI) were calculated for clinical risk factors for FFs. A multivariable logistic regression analysis was performed for the following risk factors: age, sex, ethnicity, history of diabetes mellitus, alcohol use, history of smoking, Rai stage, and diagnosis by DXA. The extent of missing data was documented, and it was assumed to be missing completely at random. A complete case analysis approach was implemented for missing data. Differences with a significance level of *P *< 0.05 were considered statistically significant.

## Results

Clinical characteristics

**Figure 1:**
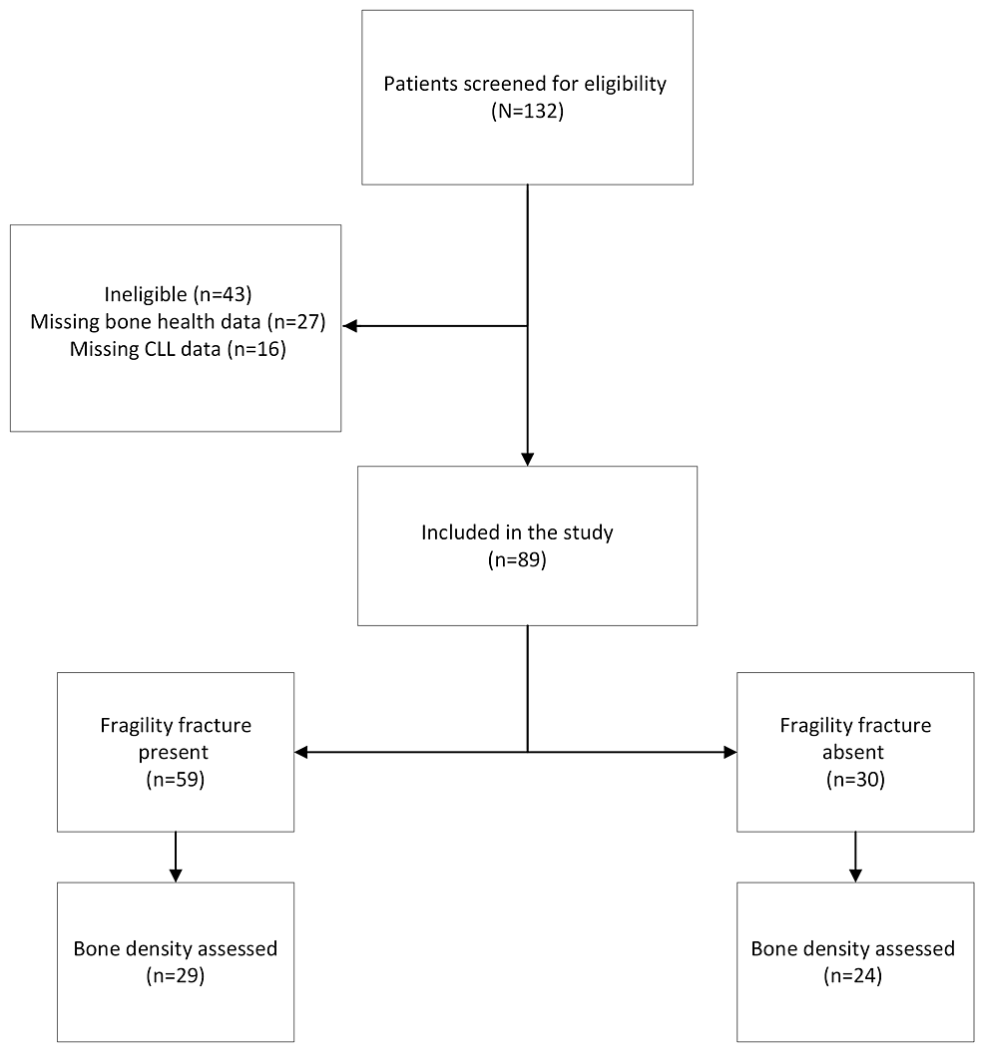
Flow diagram of cohort composition. Image credit: Lloyd Petty. CLL, chronic lymphocytic leukemia

A total of 132 patients were diagnosed with CLL and abnormal bone health, of which 43 were excluded due to missing data and 89 were included in the final analysis (Figure 1). Fifty-nine (66%) had at least one FF present (pFF) and 30 (34%) had no FFs (nFF). Participants were mainly females (*n* = 55, 62%), of Caucasian ethnicity (*n* = 83, 94%), with a mean age of 68 ± 11 years at cohort entry (Table 1). Participants with an FF were more likely to be male (pFF 54% vs. nFF 7%, *P *<= 0.01) and had a positive smoking history (pFF 36% vs. nFF 17%, *P *= 0.03).

**Table 1:** Patient characteristics at baseline and medical history, both overall and stratified by the presence or absence of fragility fractures. Note: *n* = 89. ^a^Reflects alcohol use of more than two alcohol servings a day. ^b^If information is missing from the total number of patients, the number of patients with available data on this data point is listed after the slash (/). pFF, fragility fractures present; nFF, fragility fractures absent; SD, standard deviation; DXA, dual-energy X-ray absorptiometry

Baseline characteristic	Overall (*n* = 89)	nFF (*n *= 30)	pFF (*n* = 59)
Age - years, mean (SD)			
At diagnosis of CLL	67.9 (7.7)	68.3 (16.3)	67.5 (7.8)
At diagnosis of osteoporosis	72 (5.9)	70.6 (9.4)	72.8 (9.6)
Gender			
Female, *n* (%)	55 (62)	27 (90)	28 (47)
Male, *n* (%)	34 (38)	3 (10)	31 (53)
Ethnicity			
White, *n* (%)	83 (94)	26 (87)	57 (96)
Latino, *n* (%)	1 (1)	0 (0)	1 (2)
Asian, *n* (%)	1 (1)	1 (3)	0 (2)
Native American, *n* (%)	1 (1)	1 (3)	0 (0)
Other, *n* (%)	2 (2)	2 (7)	1 (2)
Medical history			
Diabetes mellitus, *n* (%)	14 (16)	2 (7)	12 (20)
Coronary artery disease, *n* (%)	11 (12)	2 (7)	9 (15)
Rheumatoid arthritis, *n* (%)	5 (6)	3 (10)	2 (3)
Celiac disease, *n* (%)	1 (1)	1 (7)	0 (0)
Lupus, *n* (%)	1 (1)	1 (7)	0 (0)
End-stage renal disease, *n* (%)	1 (1)	0 (0)	1 (2)
Breast cancer, *n* (%)	6 (7)	6 (20)	0 (0)
Prostate cancer, *n* (%)	1 (1)	1 (3)	0 (0)
Social history			
Alcohol use, *n* (%)^a^	2 (2)	2 (6)	0 (0)
Current tobacco use, *n* (%)	3 (3)	2 (6)	1 (2)
Former tobacco use, *n* (%)	24 (27)	3 (10)	20 (34)
History of radiation, *n* (%)^b^	10/83 (12)	4/27 (15)	6/56 (11)
History of chemotherapy, *n* (%)	43 (49)	13 (43)	30 (51)
Bone mineral density (g/cm^2^), mean (SD)^b^			
Lumbar spine	0.890/33 (0.161)	0.841/15 (0.146)	0.931/18 (0.165)
Right hip	0.793/27 (0.086)	0.751/11 (0.067)	0.821/16 (0.088)
Left hip	0.780/33 (0.10)	0.766/18 (0.095)	0.797/15 (0.109)
Right femoral neck	0.658/28 (0.091)	0.624/13 (0.087)	0.687/15 (0.087)
Left femoral neck	0.640/33 (0.092)	0.626/19 (0.105)	0.660/14 (0.145)
Distal radius	0.589/23 (0.090)	0.593/9 (0.069)	0.587/14 (0.104)
Lowest *T*-score values by DXA^b^			
Lowest *T*-score: -1 or higher, *n* (%)	5/53 (9)	0	5/29 (17)
Lowest *T*-score: between -1 and -2.5, *n* (%)	20/53 (38)	7/24 (29)	13/29 (45)
Lowest *T*-score: -2.5 or lower, *n* (%)	28/53 (53)	17/24 (71)	11/29 (38)

Laboratory and CLL data 

Blood count analysis, including white blood cell count, absolute lymphocyte count, hemoglobin, and platelets, showed no significant differences between the pFF and nFF groups (Table 2). Of the 89 participants, 44 (49%) were at low risk (Rai stage 0) at presentation. However, participants with an FF were more likely to be at intermediate (Rai stages 1-2) or high risk (Rai stages 3-4) at initial presentation (pFF 58% vs. nFF 33%, *P *= 0.02). A substantial amount of data was missing for fluorescence in situ hybridization (FISH) analyses, immunoglobulin heavy chain variable region gene (IGHV) mutation, LDH, and karyotype analyses (Table 2). 

**Table 2:** Laboratory data and Rai stage at the initial presentation of individuals with CLL and decreased bone health with and without fragility fractures. ^*^Abnormality present reported. ^#^≥3 karyotype abnormalities present. CLL, chronic lymphocytic lymphoma; pFF, fragility fracture present; nFF, fragility fractures absent; WBC, white blood cell; IQR, interquartile range; Hb, hemoglobin; ALC, absolute lymphocyte count; SD, standard deviation; Vit D, vitamin D; TSH, thyroid-stimulating hormone; intmed, intermediate; LDH, lactate dehydrogenase; FISH, fluorescence in situ hybridization; IGHV, immunoglobulin heavy-chain variable region gene

	Normal range	pFF (*n *= 59)	nFF (*n *= 30)	Missing (*n*, %)	*P*-value
WBC (median, IQR)	4.3-11.3 k/µL	16.8 (11-30.9)	14.1 (8.3-24.5)	12 (13)	0.4
Hb (median, IQR)	14.8-17.8 g/dL	14 (12-15.1)	13.6 (12.7-14.1)	16 (18)	0.2
ALC (median, IQR)	>2,000 cells/mm^3^	9.7 (4.3-17.6)	10 (4.8-18.8)	26 (29)	0.7
Platelet (median, IQR)	159-439 k/µL	232 (155.8-290)	206 (177.5-262.5)	16 (18)	0.9
Calcium (mean ± SD)	8.6-1.2 mg/dL	9.4 ± 0.5	9.5 ± 0.5	11 (12)	0.2
25-Vit D (mean ± SD)	30-80 ng/mL	35 ± 13	35 ± 15	47 (53)	0.8
TSH (median, IQR)	0.27-0.42 mU/L	2 (1.2-3.0)	1.7 (1.3-2.9)	33 (37)	0.9
Rai stage (*n*, %)	-	-	-	1 (1)	0.1
Low risk: 0	-	24 (41)	20 (67)	-	-
Intmed risk: 1-2	-	24 (41)	6 (20)	-	-
High risk: 3-4	-	10 (17)	4 (13)	-	-
Elevated LDH (*n*, %)	0.8-2.4 mcg/mL	11 (19)	9 (30)	27 (30)	0.4
FISH analysis^* ^(*n*, %)	-	-	-	35 (39)	-
Del (17p)	-	4 (7)	1 (3)	-	0.6
Del (11q)	-	1 (1.7)	3 (10)	-	0.3
Del (13q)	-	20 (34)	16 (53)	-	0.4
Trisomy 12	-	7 (12)	4 (13)	-	1.0
IGHV mutation^*^	-	13 (22)	8 (27)	48 (54)	0.8
Complex karyotype^# ^(*n*, %)	-	4 (7)	4 (13)	44 (49)	0.4

CLL therapy

Forty-three (48%) participants were treated with chemotherapy (pFF 51% vs. nFF 45%, *P *= 0.5). The most common targeted and chemotherapeutic agents administered were rituximab (*n* = 18, 42%), fludarabine (*n *= 10, 23.5%), cyclophosphamide (*n *= 8, 19%), bendamustine (*n *= 7, 16%), chlorambucil (*n *= 7, 16%), and ibrutinib (*n* = 5, 12%). Out of the participants who received an initial cycle of chemotherapy (43), 21 (49%) received ≥2 cycles of chemotherapy (pFF 47% vs. nFF 54%, *P *= 0.7). Ten (9%) participants were treated with radiation therapy (pFF *n* = 6 vs. nFF *n* = 4, *P *= 0.6).

BMD and FFs

**Figure 2:**
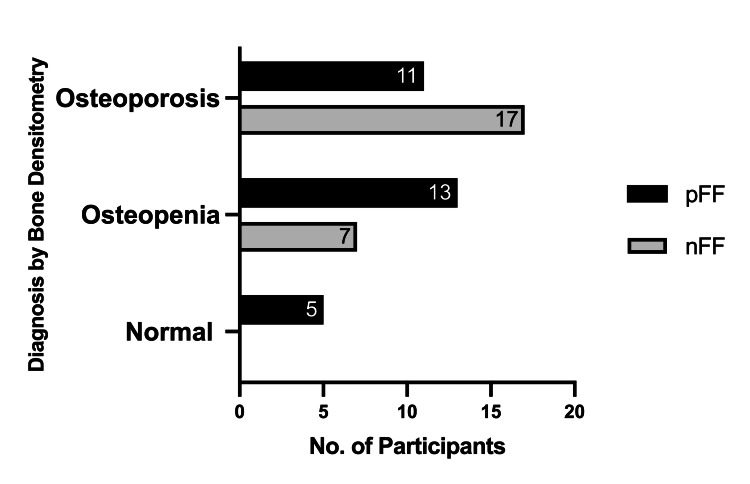
Bone densitometry diagnoses of normal bone density, osteopenia, and osteoporosis in individuals with CLL with and without fragility fractures. pFF, fragility fracture present; nFF, no fragility fracture; CLL, chronic lymphocytic leukemia

Although every patient in the study had a diagnosis of abnormal bone health, only 53 (60%) patients had DXA results (Table 3). Participants with a history of an FF were less likely to be diagnosed with osteoporosis by DXA compared to those without an FF (pFF 38% vs. nFF 71%, *P *= 0.02) (Figure 2). Similarly, participants with an FF were less likely to receive treatment for osteoporosis (pFF 41% vs. nFF 67%, *P *= 0.02). Bisphosphonates were the most frequently prescribed therapy (*n* = 89, 40%) followed by denosumab (*n *= 10, 11%). Only two participants were treated with an anabolic agent, and both had a history of FF.

**Figure 3:**
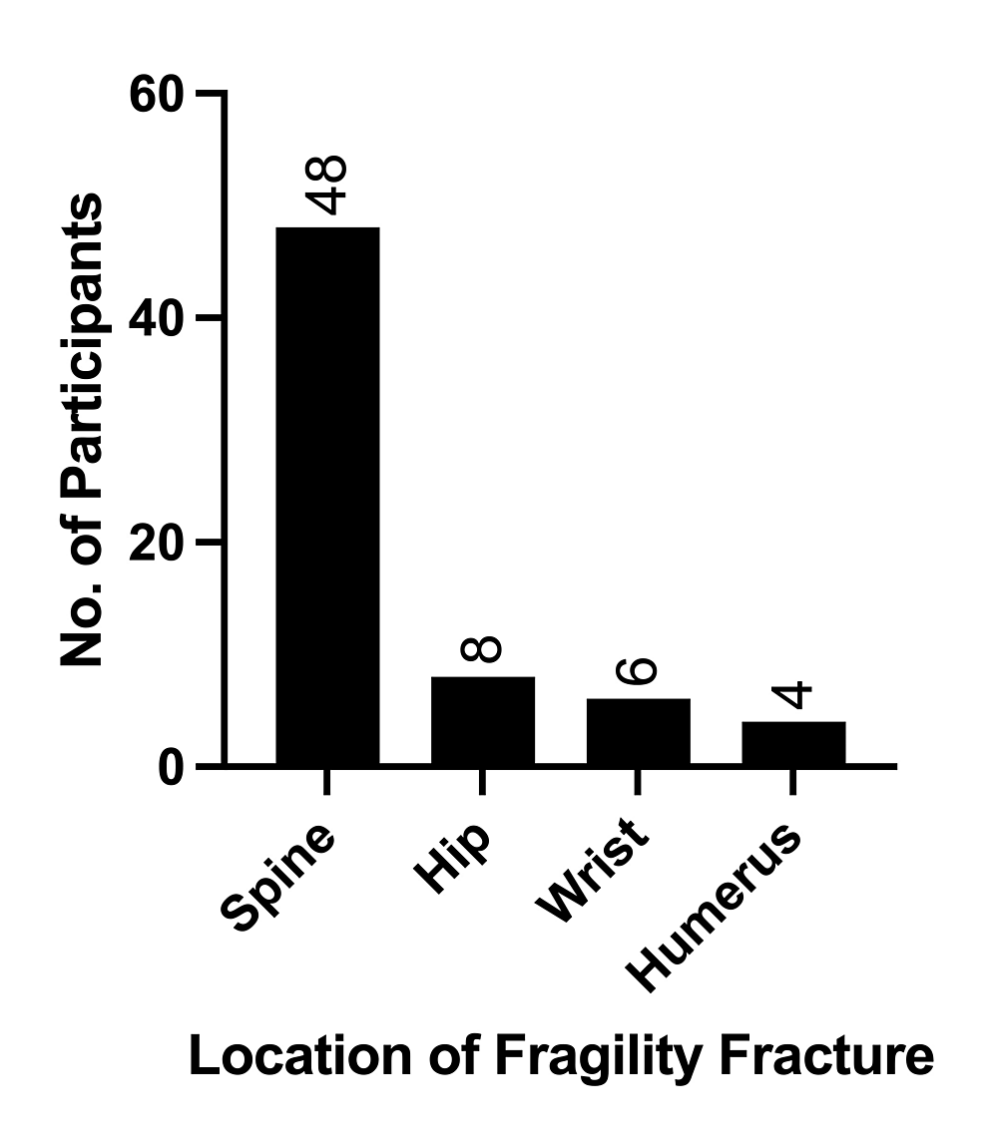
Location of fragility fractures.

**Figure 4:**
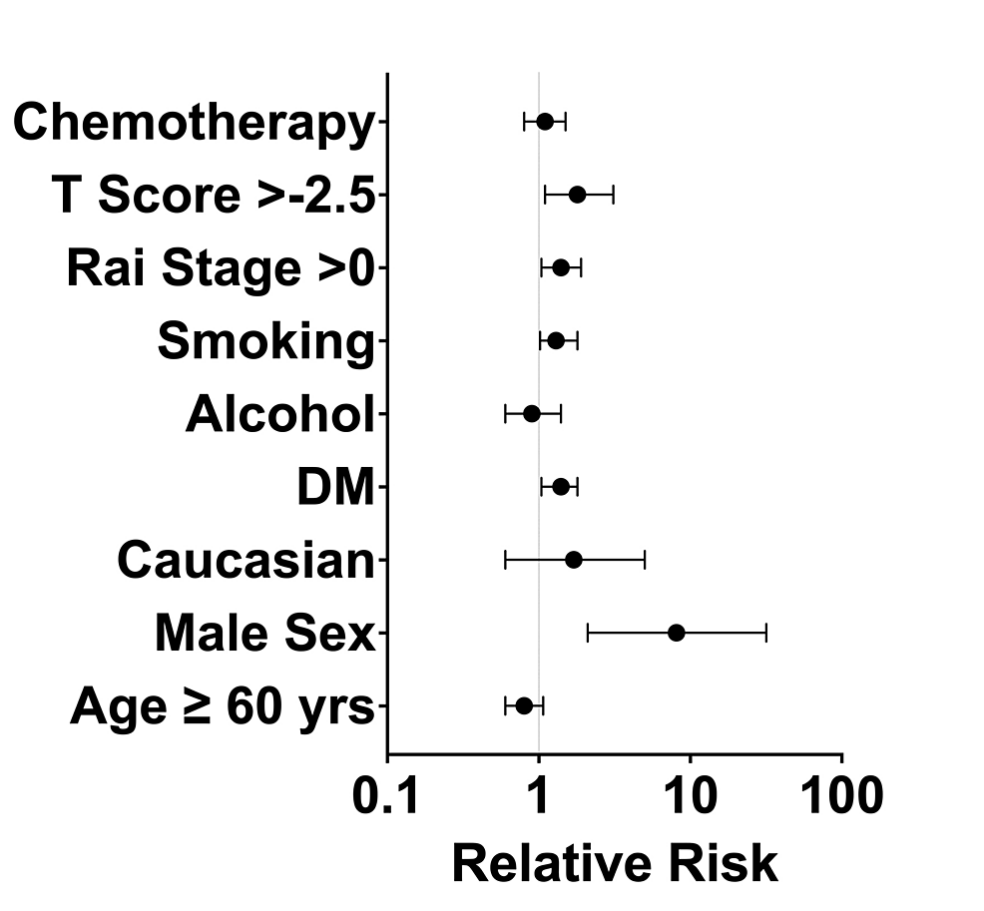
Forrest plot of relative risks (95% CI) for clinical factors associated with fragility fractures. CI, confidence interval

Of the total number of FFs, 48 (81%) were vertebral fractures (Figure 3). X-ray noted 81% (*n *= 48) of FFs, CT identified 42% (*n *= 25), and MRI revealed 24% (*n *= 14). Specifically, vertebral fractures were detected by X-ray in 79% (*n* = 38/48), CT in 44% (*n* = 21/48), and MRI in 29% (*n *= 14/48). Pertinent risk factors for FFs in individuals with CLL included male sex (RR 8.1, 95% CI 2.1-31.7), a history of diabetes mellitus (RR 1.4, 95% CI 1.04-1.8), a history of smoking (RR 1.3, 95% CI 1.02-1.8), Rai stage >0 (RR 1.4, 95% CI 1.04-1.9), and a *T*-score >-2.5 (RR 1.8, 95% CI 1.1-3.1) (Figure 4). After a multivariate analysis, male sex (*P *< 0.01) and *T-*score >-2.5 on DXA (*P *< 0.01) were significantly associated with the presence of FFs.

Follow-up

During the study period, 22% (*n *= 20) of participants were deceased, with a mortality rate of 20% in the pFF group compared to 27% in the nFF group (*P *= 0.5). Causes of death listed were sepsis (*n *= 4, 4.5%), complications of chemotherapy (*n *= 2, 2.2%), cardiac arrest (*n *= 3, 3.4%), stroke (*n *= 2, 2.2%), malignancy/CLL (*n *= 1, 1.1%), and transition to hospice or unknown (*n *= 8, 9%). There were (*n *= 2, 2.2%) cases, which involved acute bone fractures.

## Discussion

Up to two-thirds of individuals with CLL and abnormal bone health experienced FF despite most having non-osteoporotic *T*-scores. Risk factors for FF were male sex, a history of diabetes mellitus, a positive smoking history, Rai stage >0 at diagnosis, and a non-osteoporotic *T*-score on DXA. Unfortunately, 40% (*n* = 36) of our cohort did not have a DXA scan despite meeting the criteria for it based on a prior diagnosis of abnormal bone health. Furthermore, of those with FF, 51% (*n* = 29) did not undergo DXA scanning and 59% (*n *= 35) did not receive osteoporosis treatment despite meeting criteria. Taken together, this highlights a significant gap in the screening and treatment of osteoporosis in our patients with CLL.

Consistent with prior reports, the frequency of vertebral fractures is high among patients with CLL [6,7]. B-cell malignancies, including CLL, display abnormal bone remodeling with increased bone resorption and decreased formation [4]. Interestingly, the receptor activator of nuclear factor kappa beta ligand (RANKL) is expressed and released by CLL cells, contributing to the cytokine milieu related to disease activity and reduction in osteoclastogenesis [10]. As such, bone loss can be expected in people affected by CLL. Indeed, Ruchlemer et al. showed that 49% of patients with CLL had evidence of bone loss by DXA [5]. Vertebral fractures are likely more frequent due to the high trabecular bone content in the vertebrae, potentially reflecting increased disease activity found in the spine compared to other large bones. There is increased bone marrow activity in the axial skeleton in people with CLL [11]. We did show higher disease activity (Rai stage >0) was associated with the presence of FFs. Similarly, in a case-control study involving individuals aged 65 years or older with CLL, the risk of axial fractures (vertebral and pelvic) was significantly higher compared to controls (HR 1.37, 95% CI 1.25-1.50, *P *< 0.001) [6]. Male sex was also a notable risk factor for FF, in keeping with our findings. Interestingly, CLL tends to affect men more than women and the male sex is a known prognostic factor [12]. Other CLL prognostic factors, however, were not significant in our cohort.

Many demographic risk factors for CLL overlap with osteoporosis. Older age and European ethnicity are more frequent for both diagnoses [3,13,14]. Other risk factors for FF noted in our cohort are also known risk factors for osteoporosis (diabetes mellitus and smoking) [15]. In addition, the finding that osteoporotic *T*-scores were not associated with fracture risk has been shown in other hematologic populations [16]. This highlights the limitations of DXA in predicting fractures in these populations. Screening with spine imaging for vertebral fractures or the assessment of trabecular bone scores may provide more useful data in assessing fracture risk [17]. 

Our cohort met the criteria for screening/diagnostic DXA scanning based on a prior diagnosis of abnormal bone health. Unfortunately, 40% did not receive a DXA scan. In addition, the presence of FF should prompt initiating osteoporosis treatment, suggesting a lack of awareness of the diagnostic criteria for clinical osteoporosis [18,19]. While there is a focus on bone health in the American Society of Clinical Oncology (ASCO) survivorship guidelines for both breast and prostate cancer, there is no mention of any type of bone health assessment in CLL guidelines [20-24]. Incorporating a comprehensive, multidisciplinary team with a bone health expert/endocrinologist at diagnosis and during survivorship in CLL patients can likely improve overall care and quality of life [25-26].

A major strength of our study is a real-world evaluation of bone healthcare in CLL patients with abnormal bone health at a tertiary center over an almost 20-year period. We were able to determine easily identifiable clinical risk factors for FF. However, CLL patients without an associated diagnosis of abnormal bone health were not included. We, therefore, could be missing patients who truly have bone disease but are not identified by ICD codes. In addition, important CLL prognostic markers (e.g., IGHV mutation, chromosomal alterations, and beta-2 microglobulin) were not available in all patients. Additionally, patients who had an available DXA scan did not use the same machine. Finally, the effect of different chemotherapeutic agents and long-term outcomes on bone health could not be assessed.

## Conclusions

Patients with CLL and abnormal bone health exhibit a high frequency of fragility fractures, with the majority occurring in the spine. Male sex, history of diabetes mellitus, smoking, Rai stage >0, and non-osteoporotic *T*-scores are all important clinical factors that should prompt a comprehensive bone health evaluation. A larger multi-institutional study should be conducted to determine the true prevalence of FF at diagnosis of CLL and if spine imaging and trabecular bone scores should be standard of care at initial evaluation. There is a large gap in bone health assessment for our patients with CLL, highlighting the need for a more comprehensive, multidisciplinary approach to our care for those with CLL.
